# Reconciling ecology and evolutionary game theory or “When not to think cooperation”

**DOI:** 10.1073/pnas.2413847122

**Published:** 2025-03-31

**Authors:** Corina E. Tarnita, Arne Traulsen

**Affiliations:** ^a^Department of Ecology and Evolutionary Biology, Princeton University, Princeton, NJ 08544; ^b^Department of Theoretical Biology, Max Planck Institute for Evolutionary Biology, Plön 24306, Germany

**Keywords:** evolutionary game theory, replicator dynamics, ecological interactions, intrinsic growth rates, adaptive dynamics

## Abstract

Evolutionary game theory (EGT)—overwhelmingly employed today for the study of cooperation in various systems, from microbes to cancer and from insect to human societies—started with the seminal 1973 paper by Maynard Smith and Price showing that limited animal conflict can be selected at the individual level. Owing to the explanatory potential of this paper and enabled by the powerful machinery of the soon-to-be-developed replicator dynamics, EGT took off at an accelerated pace and began to shape expectations across systems and scales. But, even as EGT has expanded its reach, and even as its mathematical foundations expanded with the development of adaptive dynamics and inclusion of stochastic processes, the replicator equation remains, half a century later, its most widely used equation. Owing to its early development and its staying power, the replicator dynamics has helped set both the baseline expectations and the terminology of the field. However, much like the original 1973 paper, replicator dynamics rests on the assumption that individual differences in reproduction are determined only by the payoff from the game (i.e., in isolation, all individuals, regardless of their strategy, have identical intrinsic growth rates). Here, we argue that this assumption limits the scope of replicator dynamics to such an extent as to warrant not just a more deliberative application process, but also a reconsideration of the broad predictions and terminology that it has generated. Simultaneously, we reestablish a dialog with ecology that can be mutually fruitful, e.g., by providing an explanation for how diverse ecological communities can assemble evolutionarily.

Game theory as a mathematical discipline was founded by von Neumann at the beginning of the 20th century and quickly established itself as a relevant perspective on economic behavior (1). It was not long before biologists realized that a game theoretic perspective might also be fruitful in biology. By the early 1960s, Lewontin suggested that “the modern theory of games may be useful in finding exact answers to problems of evolution not covered by the theory of population genetics” (2) and Slobodkin contemplated an analogy between his experimental findings on interspecific interactions and the result of species playing games with the environment (3). These early attempts showed foresight, but they relied either on imprecise verbal analogies or on unwieldy mathematical formulations. It would be another decade before Maynard Smith and Price (4) achieved the trifecta of i) a compelling and elusive question, with ii) a simple, tractable, and biologically meaningful game theoretic formulation that yielded iii) an insightful and biologically coherent answer.

Prior to Maynard Smith and Price (4), explanations for limited conflict—the apparent reluctance of animals to engage in “total war” that could result in severe injuries or death—were grounded in a “species-selection” view, whereby total war would be bad for the species. Maynard Smith and Price employed game theoretic thinking to explore strategies that males of the same species might employ during conflict; the two that would become most famous for illustrating the power of the new framework were Hawk (aggressive males that fight to death or until serious injury) and Dove [pacifist males that backed down from conflict; originally called “Mouse” (4)]. The game matrix

[1] $$\begin{array}{c} \begin{array}{cc} & \begin{array}{cc} H & D \end{array}\\ \begin{array}{c} H\\ D \end{array} & \left(\begin{array}{cc} (b-c)/2 & b\\ 0 & b/2 \end{array}\right) \end{array} \end{array}$$

captures with fewest parameters the expected payoff obtained in a pairwise encounter: Two Doves would split the benefit *b* > 0, a Dove encountering a Hawk would retreat, getting payoff 0 and yielding the entire benefit to the Hawk, and two Hawks would fight, resulting in an expected payoff of (b−c)/2<0, where c>b>0 to reflect the high cost of total war.

Under the assumption that the only difference in fitness between males of the same species is the conflict strategy employed, Maynard Smith and Price investigated whether either of these strategies would be evolutionarily stable (ESS). For a strategy to be evolutionarily stable, it must be resistant to invasion by rare mutants (4). Applying this concept to the game matrix above, the Hawk strategy is ESS if its payoff playing against itself exceeds the payoff that a rare mutant Dove strategy gets from playing against the resident Hawk. This analysis revealed that neither Hawk nor Dove is ESS and that the higher the cost of war, the worse a population of all Hawk fares. From these results, one can intuit that, since neither strategy is ESS, invaders of either type will grow into a resident population of the other type, but not indefinitely. The population should thus equilibrate at a mix of the two strategies, with the proportion of Hawk decreasing with the magnitude of the cost relative to the benefit. That sufficed for Maynard Smith and Price to demonstrate that selection at the individual level does not favor a full Hawk population (i.e. “total war”), but a mix of some aggressive individuals with mostly pacifist ones.

Because this paper revealed such enormous potential for game theory—and its fundamental underlying idea that the success of any given strategy should depend on the frequencies of all strategies in a population—to bring clarity and alternative hypotheses to elusive questions in animal behavior, mathematical biologists (5–7) quickly began to develop a dynamical theory that allowed the exploration of the full strategy space, while recapitulating the outcomes of the ESS approach in the special case of the homogeneous equilibria. This framework became known as the replicator dynamics, in which the replicator equations giving the change in the frequency of strategy *i* with time took the simplest form, ${\dot{x}}_{i}=x_{i}\left(f_{i}-\overset{¯}{f}\right)$. Here—much as R.A. Fisher had proposed earlier for constant selection (8)—the growth rate is given by the difference between *f*_*i*_, the fitness that an individual using strategy *i* derives from the game (which depends on the matrix entries and on the frequency of each strategy in the population), and $\overset{¯}{f}=\sum_{i}x_{i}f_{i}$, the average population fitness.

Mathematically elegant and dynamically rich, the replicator dynamics quickly established itself as the formalism of evolutionary game theory, and its straightforward and intuitive predictions set both the baseline expectations (i.e. evolutionary outcomes for a large, well-mixed population) and the terminology of the field (Fig. 1): cooperators and defectors engage in a Prisoner’s Dilemma that results in the dominance of defectors and demise of cooperators; Hawk- and Dove-type strategies that benefit from each other’s presence result in coexistence; and coordination games (e.g., Stag-Hunt) between strategies that do worst when mixed together result in bistability.

**Fig. 1.**
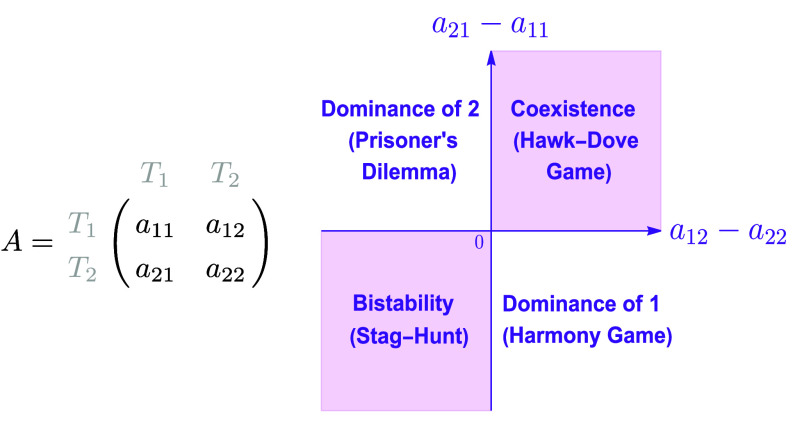
Classification of general 2 × 2 games. Matrix *A* captures the interaction between two types, $T_{1}$ and $T_{2}$; i.e., *a*_*ij*_ is the effect that type *j* has on the fitness of type *i*. The replicator dynamics produces a two-dimensional game classification depending on the sign of the two terms $a_{21}-a_{11}$ and $a_{12}-a_{22}$. Without loss of generality, we assume that $a_{11}>a_{22}$, such that the *Upper Left* quadrant captures Prisoner’s Dilemma Games.

Although dynamically richer than the original ESS analysis, the replicator dynamics was also based on the assumption that the only difference in the reproductive ability of different strategies comes from differential payoffs within the game itself, an assumption that is unlikely to be generally true. During the 1990s, adaptive dynamics (9–11) was developed to generalize the fundamental idea of frequency-dependent interactions by coupling it with population dynamics, thereby relaxing the assumption that the game is solely responsible for fitness differences. Adaptive dynamics has since successfully tackled challenging evolutionary questions involving quantitative (continuous) traits, where any mutant that arises is only slightly different from the resident (12). But the applicability of the replicator dynamics to both continuous and discrete traits and to mutations of any effect size, its higher mathematical tractability, and the priority effect of its simple, accessible predictions having, in large part, established the conceptual terminology and expectations of the field since its inception have all contributed to its remarkable endurance.

The staying power of replicator dynamics—“the first explicit differential equation modeling the frequencies of strategies in a population, and still today the most widely used” (13)—has had both theoretical and empirical implications. Theoretically, the assumption of the game as sole determinant of fitness differences has been further perpetuated as EGT has been generalized to address, for example, demographic stochasticity (14–16) or spatial structure (17). The lack of an explicit theoretical reckoning with the biological consequences of such a major assumption—even as EGT expanded its reach from animals to microbes (18–22) and from microbes to cancer (23–25)—has led to predictions of replicator dynamics being applied broadly to systems where the original assumption of the game as sole determinant of fitness differences might not hold (26, 27). Here, we attempt such a reckoning and ask: How would the baseline expectations of EGT have differed had the original formulation of both ESS and replicator dynamics accounted for the possibility of intrinsic fitness differences that are independent of the game?

## More than Just the Game

As has been the case both for replicator dynamics and for adaptive dynamics, we continue to assume that mutations are rare, i.e. that the resident population is at a dynamical equilibrium when a new mutant arises. This assumption—itself a constraint whose removal has led to exciting work (28)—rests on the expectation that evolutionary and ecological timescales are substantially different and that, once a mutant arises, the ecological dynamics takes over. Therefore, to answer our question, we start from the generalized Lotka–Volterra equations, the simplest extension that accounts not just for interactions with one’s own type and others, but also for the possibility of different intrinsic growth rates *r* (i.e. a type-specific growth rate when rare, in the absence of any interactions). The generalized Lotka–Volterra equations capture the dynamics of absolute abundances, *n*_*i*_, as ${\dot{n}}_{i}=n_{i}\left(r_{i}+\sum_{j}a_{\mathrm{ij}}n_{j}\right)$, where *a*_*ij*_ is the density-dependent effect^*^ of type *j* on type *i*.

For simplicity of exposition, we will restrict ourselves to two types, but the relationship between the two frameworks derived below in Eq. **3** can be generalized to any number of types and raises qualitatively the same issues (*SI Appendix*). For two types interacting according to the general matrix *A* in Fig. 1, where *a*_*ij*_ is the effect that a type *j* individual has on the growth rate of a type *i* individual, we can write the Lotka–Volterra equations to track the dynamics of their abundances:

[2] $$\begin{array}{c} \begin{array}{cc} {\dot{n}}_{1} & =n_{1}\left(r_{1}+a_{11}n_{1}+a_{12}n_{2}\right)\\ {\dot{n}}_{2} & =n_{2}\left(r_{2}+a_{21}n_{1}+a_{22}n_{2}\right) \end{array} \end{array}$$

From the dynamics of abundances, we can use the quotient rule to write the standard mathematical derivation for the dynamics of relative frequencies, $x_{1}=n_{1}/\left(n_{1}+n_{2}\right)$ and $x_{2}=n_{2}/\left(n_{1}+n_{2}\right)$, to permit the comparison with replicator dynamics. We obtain:

[3] $$\begin{array}{c} {\dot{x}}_{1}=\left(n_{1}+n_{2}\right)\left[x_{1}x_{2}\left(\frac{r_{1}-r_{2}}{n_{1}+n_{2}}+f_{1}-f_{2}\right)\right], \end{array}$$

where we have denoted $f_{1}=a_{11}x_{1}+a_{12}x_{2}$ and $f_{2}=a_{21}x_{1}+a_{22}x_{2}$ (see *SI Appendix* for the step-by-step derivation). The equation for $x_{2}$ can be similarly derived, but it is redundant since $x_{2}=1-x_{1}$.

This is a crucial point in the derivation. The positive factor $n_{1}+n_{2}$ in Eq. **3** simply rescales the dynamics but is irrelevant to the outcome; the relevant term is the one in the square brackets.

- If the fraction inside the brackets is zero—either because the two types have the same intrinsic growth rates (i.e. $r_{1}=r_{2}$ and thus fitness differences arise only from the game) or because the population grows without bounds (i.e. $n_{1}+n_{2}\to \infty$), then Eq. **3** is dynamically identical (up to a rescaling of time by the positive factor $n_{1}+n_{2}$) to the equation
  [4] $$\begin{array}{c} {\dot{x}}_{1}=x_{1}x_{2}\left(f_{1}-f_{2}\right). \end{array}$$
  This equation is precisely the replicator equation ${\dot{x}}_{1}=x_{1}\left(f_{1}-\overset{¯}{f}\right)$ for two types playing the game given by matrix *A* in Fig. 1, where $f_{1}=a_{11}x_{1}+a_{12}x_{2}$ is the fitness of an individual of type 1 and $\overset{¯}{f}=x_{1}f_{1}+x_{2}f_{2}$ is the average population fitness. Note that, although a priori it might seem like the interaction terms in a Lotka–Volterra system should have different units than those in the replicator dynamics (specifically, 1/(t×number) versus 1/t), the nondimensionalization is accounted for by the dynamical rescaling term $n_{1}+n_{2}>0$, so that Eqs. **2** and **4** are, indeed, written for the same matrix *A* given in Fig. 1.
- If, however, the system is bounded and the two types have different intrinsic growth rates, the fraction inside the brackets in Eq. **3** can be nonnegligible.^†^ In that case, the dynamics of frequencies cannot be separated from the dynamics of abundances and, thus, Eq. **3** is not a stand-alone equation for the dynamics of frequencies (since solving it requires the equations for $dn_{i}/dt$). In other words, in a bounded system where differences in fitness exist even in the absence of the game, Eq. **3** is not a more general replicator equation; in this case, a replicator equation does not exist.

This derivation—and its general form for *m* types shown in *SI Appendix*—recapitulates the equivalence established many decades ago (29–31) between the *m*-type Lotka–Volterra system with identical intrinsic growth rates and the replicator dynamics for an *m*-type game. But it also serves two additional ends. First, it explicitly highlights the conditions that are both necessary and sufficient for the equivalence, thereby allowing us to engage in depth with the scenarios where those conditions are violated. Specifically, if the types have different intrinsic growth rates, i.e. the game is not the sole determinant of fitness differences, the outcome of their dynamics cannot be predicted with the replicator equation.^‡^ This remains true even if the ecological interactions are frequency, rather than density, dependent (*SI Appendix*). In other words, in the general case when differences in fitness can arise even in the absence of the game, the basic expectations for what would happen in the defining games of EGT (Fig. 1) could change. This is because the higher intrinsic growth rate *r*_*i*_ of a certain strategy *i* could compensate (up to a point) even for negative between-strategy game terms $\sum_{j}a_{\mathrm{ij}}x_{j}$, thus possibly leading to survival of a “losing” game strategy. Then, applying the predictions of replicator dynamics (Fig. 1) broadly to empirical systems that it was not designed to study has, at best, high potential for confusion.

**Fig. 2.**
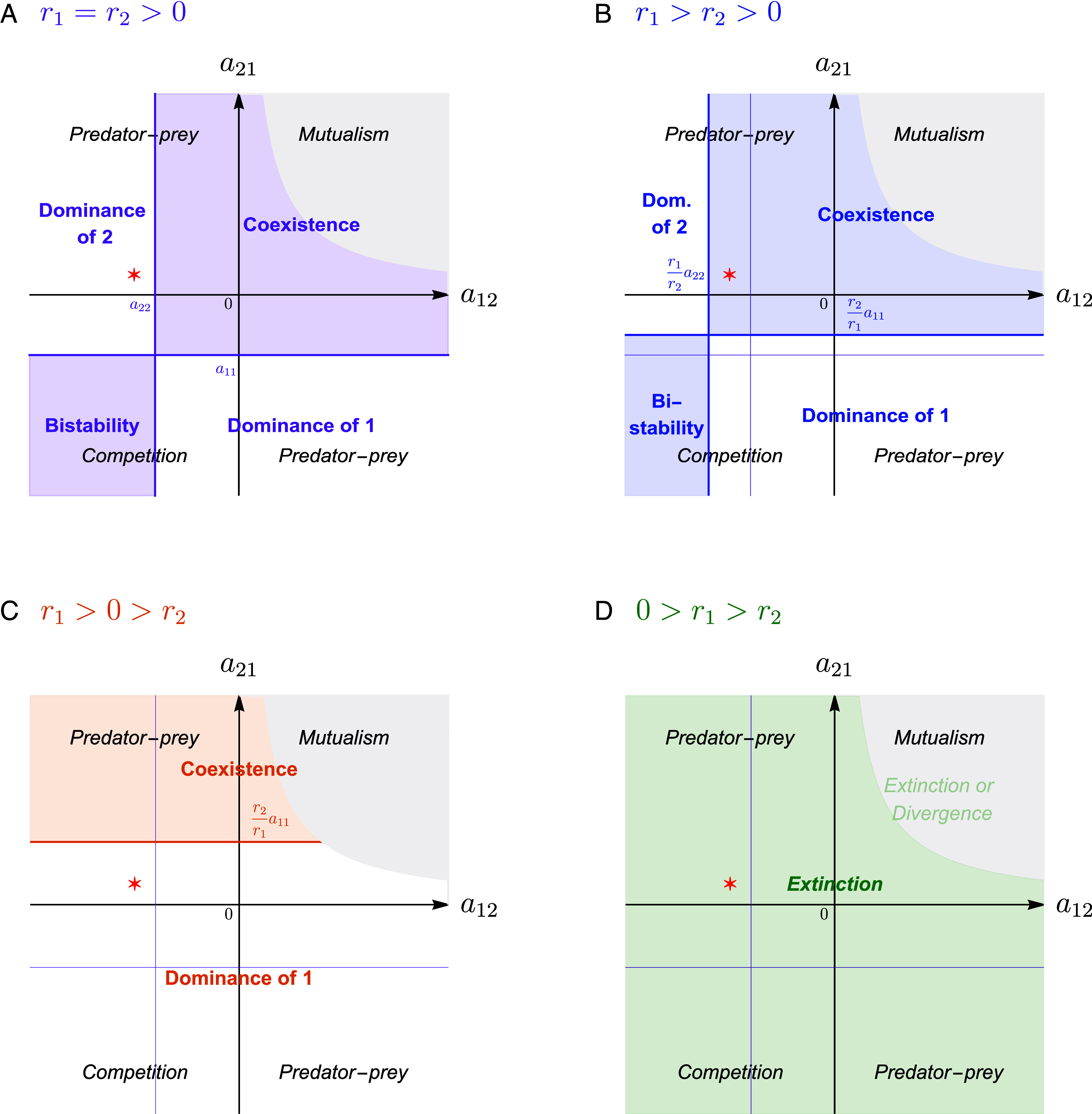
Expanded worldview. Relationship between ecological classification of interaction type [black axes, (*A*–*D*)], game theoretical classification of the dynamics under the assumption that $r_{1}=r_{2}$ [in purple, (*A*–*D*); corresponding to Fig. 1], and correct dynamical classification when $r_{1}\neq r_{2}$ [(*B*), in blue; (*C*), in orange; and (*D*), in green]. In the gray areas, $a_{12}a_{21}>a_{11}a_{22}$ and the system is unbounded and outside the scope of our study. (*A*) When $r_{1}=r_{2}>0$, the replicator dynamics (RD) is equivalent to Lotka–Volterra (LV) and thus predicts accurately the four possible dynamical outcomes of the ecological interactions (which differ from the ecological classification into interaction types; e.g., not all competitive interactions result in bistability). (*B*–*D*) When $r_{1}\neq r_{2}$, the RD does not accurately predict the outcome of the ecological interactions and the game theoretical classification loses its meaning. Correctly predicting dynamics using LV translates the axes of the purple coordinate system to account for growth rates, in addition to interaction terms (compare blue, orange, green versus purple coordinate systems). The red star in (*A*–*D*) marks an interaction matrix that is a generalized Prisoner’s Dilemma game when the growth rates are equal (*A*); see Fig. 3 for a full analysis of this example. Parameters: $a_{11}=-3$, $a_{22}=-4$, $r_{1}=r_{2}=1$ in (*A*), $r_{1}=1.5$ and $r_{2}=1$ in (*B*), $r_{1}=1$ and $r_{2}=-1$ in (*C*), and $r_{1}=-0.5$ and $r_{2}=-1$ in (*D*); Red star: $a_{12}=-5$ and $a_{21}=1$.

Second, the derivation allows a useful comparison. Although EGT has been overwhelmingly applied to types of the same species, analogies between strategic interactions, such as cooperation, and species interactions, such as mutualism, have unsurprisingly arisen (32–36). Therefore, the fact that the generalized LV are precisely the equations used by ecologists to catalog ecological interactions presents the added benefit of allowing a direct terminology comparison between strategy interactions and species interactions. To this end, the black and white plot throughout Fig. 2 illustrates the three main ecological interactions that matrix *A* in Fig. 1 captures depending on the signs of the interspecific parameters ($a_{12}$, $a_{21}$): mutualism (+,+), predator–prey or parasitism (+,− or vice versa), and competition (−,−).

## How Different Is the Worldview If the Game Is Not Necessarily the Sole Determinant of Fitness Differences?

To catalog the possible discrepancies, we consider a bounded system: For this, either type in isolation must be bounded (i.e. $a_{\mathrm{ii}}<0$ for i=1,2) and the system with both types present must also be bounded (i.e. $a_{12}a_{21}<a_{11}a_{22}$). Without loss of generality, we assume that type 2 experiences the stronger intratype competition, i.e. $a_{11}>a_{22}$ (equivalently, that type 1 gets a higher payoff against itself than type 2 does against itself).

When $r_{1}=r_{2}$, we showed above that the replicator dynamics given by Eq. **4** is dynamically equivalent to the Lotka–Volterra system and, thus, dynamical outcomes of LV are accurately captured by the game-type classification (17, 37, 38) (shown by the purple plot in Fig. 2*A*, which is the same as the purple plot in Fig. 1): dominance of either type 1 or type 2, coexistence, or bistability. Because we assumed that $a_{11}>a_{22}$, when the types are strategies and type 2 dominates it can be seen as a social dilemma (e.g., Prisoner’s Dilemma); when type 1 dominates, it is sometimes called “harmony.” Ecologically, this agrees with the fact that all mutualisms, some predator–prey interactions, and some competitive interactions are expected to lead to coexistence of the two species (39). The remaining predator–prey interactions can lead only to dominance, i.e. the predator drives the prey extinct; and the remaining competitive interactions lead to bistability. Conversely, this overlaying of ecological and game theoretic worldviews also reveals that a Prisoner’s Dilemma is ecologically a predator–prey (parasitic) or competitive interaction, a coordination game is a competitive interaction, while a coexistence game (such as Hawk–Dove or Snowdrift) can be any type of ecological interaction—mutualistic, competitive, or predator–prey—depending on the sign of the payoffs that each type gets from the other. Importantly, though mutualisms are occasionally discussed as the species-level analog of within-species cooperative interactions in the sense of game theory (32–36), this analysis shows that their appropriate analog are, in fact, coexistence games.

When $r_{1}\neq r_{2}$, writing the replicator dynamics solely based on the interaction matrix, and without regard to the difference in intrinsic growth rates, fails to accurately predict the dynamics of the two-type Lotka–Volterra system. This can be seen by comparing the purple system of coordinates against the blue one in Fig. 2*B*, the orange one in Fig. 2*C*, and the green one in Fig. 2*D*: The purple system captures the dynamics of Eq. **4**, as in Fig. 2*A*, whereas the other three coordinate systems capture the dynamics of the Lotka–Volterra system with the same interaction matrix but accounting for $r_{1}\neq r_{2}$. When $r_{1}>r_{2}>0$ (blue in Fig. 2*B*), the region of coexistence is shifted up and to the left, so that, for example, more cases of predator–prey interactions that used to result in the extinction of species 1 (prey) now yield coexistence (compare the red star in Fig. 2 *A* and *B*). If $r_{1}>0>r_{2}$ (orange classification in Fig. 2*C*) or $0>r_{1}>r_{2}$ (green in Fig. 2*D*), a game theoretic analysis neglecting the difference in intrinsic growth rates gets not just the precise regions corresponding to each possible outcome wrong, but also the set of possible outcomes. Specifically, when $r_{1}>0>r_{2}$ only coexistence or dominance of type 1 is possible (but not bistability or dominance of type 2), while when $0>r_{1}>r_{2}$ only extinction is possible. The shift in—and reclassification of—dynamical outcomes induced by $r_{1}\neq r_{2}$ is simple and elegant in its own right. For instance, if $r_{1}>r_{2}>0$, then the horizontal line gets moved vertically from $a_{11}$ to $a_{11}\left(r_{2}/r_{1}\right)$ and the vertical line gets moved horizontally from $a_{22}$ to $a_{22}\left(r_{1}/r_{2}\right)$. Thus, coexistence occurs if $a_{21}>a_{11}\left(r_{2}/r_{1}\right)$ and $a_{12}>a_{22}\left(r_{1}/r_{2}\right)$, bistability occurs when both inequalities are reversed, and dominance occurs when the two inequalities have different signs (Fig. 2*B* blue).

Thus, accounting for intrinsic growth rates does produce a substantially altered worldview relative to the game-type classification and associated predictions and terminology from evolutionary game theory (Fig. 1). The graphical representation in Fig. 2 reveals that changing the intrinsic growth rates relative to each other allows one to transform the dynamical outcome of any game: e.g., dominance of type 2 can be transformed into coexistence, bistability, or even dominance of type 1, by increasing the growth rate of type 1 relative to type 2 (see *SI Appendix*, Fig. S1 for a summary of all possible transformations). Such exhaustive transformations yield the existing game theoretical classification largely uninformative.

## Ceci n’est pas un... Prisoner’s Dilemma

A transformation that is particularly pertinent to our main point is the one from dominance of type 2 to coexistence. This means that an apparent Prisoner’s Dilemma game can result in coexistence if cooperators have an intrinsic growth rate advantage relative to defectors.

**Fig. 3.**
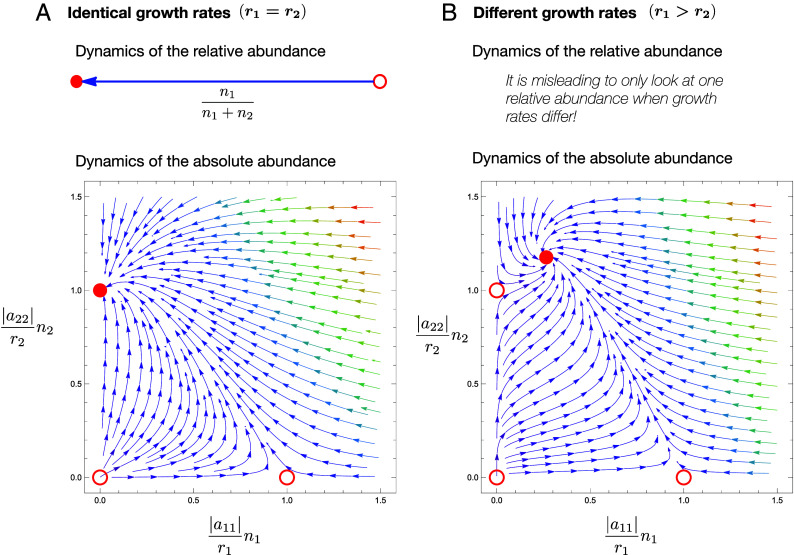
The dynamics of relative and absolute abundances for a general Prisoner’s Dilemma matrix. (*A*) When growth rates are identical, $r_{1}=r_{2}$, the relative abundance is sufficient to capture the dynamics. Here, strategy 1 is cooperation and strategy 2 is defection. The only stable equilibrium (red disk) is defection: $x_{1}=0$, $x_{2}=1$ for relative abundances, and the corresponding $\left(n_{1},n_{2}\right)=\left(0,-r_{2}/a_{22}\right)$ for absolute abundances. Unstable equilibria are marked by red circles. (*B*) When growth rates are different, $r_{1}=2$ and $r_{2}=1$, looking at relative abundances alone is insufficient. In this case, the dynamics has three unstable fixed points at the boundaries, and a new and stable fixed point emerges in the interior. The term Prisoner’s Dilemma has no meaning anymore. Interaction parameters correspond to the red star in Fig. 2: $a_{11}=-3$, $a_{12}=-5$, $a_{21}=+1$, and $a_{22}=-4$.

A Prisoner’s Dilemma is characterized by the payoff ranking $a_{21}>a_{11}>a_{22}>a_{12}$. The dilemma rests in the fact that type 2 is always better off in any pairwise interaction (owing to $a_{21}>a_{11}$ and $a_{22}>a_{12}$), but a homogeneous population of type 1 is better off than a homogeneous population of type 2 (owing to $a_{11}>a_{22}$). Thus type 1 are the cooperators and type 2 are the defectors or free-riders. The replicator Eq. **4** for the dynamics of the frequency of cooperators reveals that cooperators will always decrease in abundance and that $x_{1}=0$ (all defectors) is the only stable fixed point (Fig. 3*A*, *Top*). The dilemma therefore leads to the demise of cooperators. This prediction has set the baseline expectation for the field of EGT, with decades of work focused on uncovering mechanisms that can change the tragic outcome of the Prisoner’s Dilemma (40).

As expected, if $r_{1}=r_{2}$, the Lotka–Volterra equations predict the same outcome, with the sole stable equilibrium being the defectors-only one (Fig. 3*A*, *Bottom*). If, however, the two types have different but positive growth rates, so that $r_{1}>r_{2}>0$, we can use the graphical illustration in Fig. 2*B* to determine when the outcome of a Prisoner’s Dilemma matrix can change from dominance to coexistence, which is when $a_{12}>a_{22}\left(r_{1}/r_{2}\right)$. Without loss of generality, we can assume $r_{2}=1$. Keeping in mind that $a_{22}<0$, the condition $a_{12}>a_{22}\left(r_{1}/r_{2}\right)$ is equivalent to $r_{1}>a_{12}/a_{22}$. For example, if the interaction matrix is $a_{11}=-3$, $a_{12}=-5$, $a_{21}=+1$, and $a_{22}=-4$ (corresponding to the red star in Fig. 2), the above analysis predicts that the two types will coexist provided that $r_{1}>5/4$. In other words, in this example, cooperators and defectors can coexist, as long as the intrinsic growth rate of cooperators is 25% greater than that of the defectors. This is, indeed, exactly what we see in Fig. 3*B*.

In this case, although the matrix obeys the inequalities of a Prisoner’s Dilemma, the system is not trapped in a dilemma at all. Therefore, from a game theoretical perspective it becomes problematic to continue to argue that the types are engaged in a Prisoner’s Dilemma. In this sense, cooperation becomes less of a puzzle; if all examples of cooperation in living systems were to fall into this category, then much ink would have been spilled over a nonexistent dilemma. Of course, this is unlikely to be the case, but we are exaggerating here to paint the clearest picture of the consequences of a mathematical framework setting the expectations and terminology of a field. We would go as far as to argue that, in this case, it would even be misleading to continue to call the two types cooperators and defectors, as this nomenclature is immediately evocative of a framework that ceases to apply when the two growth rates are not identical.

## Case in Point: Invertase Production in the Yeast Saccharomyces cerevisiae

To exemplify our concern, we consider an empirical example. In a sucrose medium, yeast produces invertase to hydrolyze the sucrose into glucose, a preferred nutrient. Because invertase is costly to produce and 99% of its benefits diffuse away before they can be imported into the producing cell (26)—and are thus freely available to others—invertase has been labeled a costly public good and invertase production a cooperative behavior (21). The cooperator label naturally led empiricists to invoke the predictions of evolutionary game theory and hypothesize that the outcome of a well-mixed interaction between producers and nonproducers should be a tragedy of the commons, i.e. invertase-producers should be outcompeted by nonproducers; instead, producers and nonproducers were found to stably coexist in lab experiments (26). An important reason is that when invertase-producing yeast is rare and in a sucrose-only medium, its ability to privatize 1% of the glucose it transforms gives it a substantial intrinsic growth advantage over the knockout nonproducing strain under identical conditions (26) [see also subsequent work that has continued to uncover features of both the privatization process and other competitive advantages of producers (41, 42)]. In other words, producers and nonproducers do not have identical intrinsic growth rates in a sucrose-only medium. In this light, the coexistence outcome (26) is only puzzling and in dissonance with the theory because the theory does not directly apply to this system. If, instead, the departure point had been the Lotka–Volterra framework, the starting theoretical hypothesis would have been that the mix of the two strains will result *either* in tragedy of the commons *or* in coexistence, depending on how much higher the intrinsic growth rate of producers was than that of nonproducers (Fig. 1*B*). Lotka–Volterra, itself a phenomenological framework, would not have provided *the* correct outcome, but a richer set of outcomes within which the experimental results would have naturally existed. This ultimately interesting study of diverse microbial consumer–resource interactions illustrates the pitfalls of a deceptively intuitive terminology (“cooperation” and “costly public goods”) evocative of the deceptively broad predictions of the replicator dynamics framework. And invertase production is by no means an isolated example; other costly extracellular products have been painted with the broad brush stroke of cooperation, public goods, and the tragedy of the commons when the reality is, in fact, more ecologically nuanced [see, e.g., iron scavenging and siderophore production (43–45), where there is, similarly, some degree of privatization].

How invertase production in yeast should ultimately be modeled to derive the most out of a modeling exercise is beyond the scope of this perspective. But, it is worth mentioning that work in the wild yeast *Saccharomycesparadoxus* (46) bolsters the proposition that a richer ecological framework—in this case one that accounts for adaptation to different sucrose-availability substrates—is more appropriate for probing the natural variation in invertase production than the social conflict framework, and suggests that invertase production is a quantitative trait, making adaptive dynamics a good candidate approach. Moreover, because experimentally there is a clear and quantifiable consumer/producer-resource dynamic, models that explicitly capture that dynamic (47) can incorporate some of the more mechanistic findings (e.g., ref. 42) and likely make testable predictions that can inform broader ecoevolutionary models for the study of natural variation.

## Broader Implications: Assembling Diverse Communities, Step-by-Step

Our analysis also has fundamental implications beyond two-type interactions. Specifically, the realization that different growth rates can transform apparent dominance outcomes into coexistence of types unlocks an enormous potential for conceptualizing the assembly of ecologically complex communities. For example, cyclical dynamics have been occasionally found to exist empirically (19, 48, 49), despite skepticism from theoreticians who have pointed out that it is very hard for such cyclical interactions to be assembled, one mutant (or one migrant) at a time (50–52). Intuitively, that is because cyclical interactions exist by definition only if all types are present; if one of the types is not present yet, then the broken cycle is a dominance hierarchy that leads to competitive exclusion. However, this intuition only holds when all growth rates are identical and the dynamical outcome is entirely predicted by the replicator equation (53). When the intrinsic growth rates of at least some of the types are different, the apparent dominance hierarchies can disappear and coexistence could be built step-by-step.

**Fig. 4.**
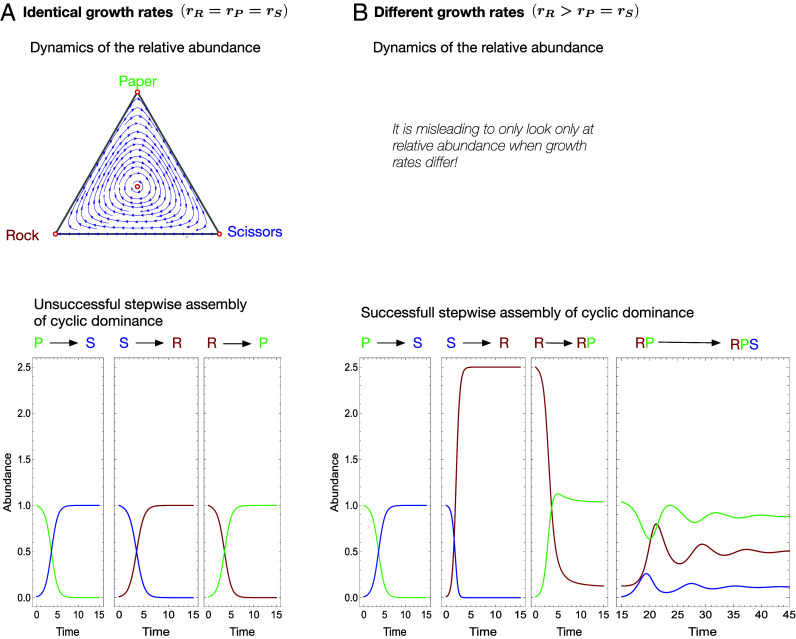
Assembling cyclic dynamics. (*A*) (*Top*) For identical growth rates, the dynamics of the relative abundance shows cyclic motion on closed trajectories around a neutrally stable fixed point. (*Bottom*) One strategy can invade one other and be invaded by the third, such that a stepwise assembly of cyclic dominance via coexistence is not possible (*Bottom*) if mutants (or migrants) are rare. (*B*) (*Top*) For asymmetric growth rates, it is misleading to consider the dynamics of relative abundances. (*Bottom*) A stepwise assembly via the coexistence of two strategies (in this case, *R* and *P*) into cyclic dominance of three types is possible. Interaction parameters: $a_{\mathrm{ij}}=-1\pm \alpha$ with *α* = 1.3; growth rates: $r_{R}=r_{P}=r_{S}=1$ in (*A*) and $r_{P}=r_{S}=1$, $r_{R}=2.5$ in (*B*).

In *SI Appendix* and Fig. 4 and *SI Appendix*, Fig. S2 we exemplify how this would work in the simplest case of cyclical dynamics, that of Rock-Paper-Scissors. We work with the simplest game matrix (i.e., completely symmetric, having a single parameter *α* > 0, such that winning leads to +α and losing to −α) that also incorporates the assumption of bounded growth for each individual type:

[5] $$\begin{array}{c} \begin{array}{cc} & \begin{array}{ccc} R & P & S \end{array}\\ \begin{array}{c} R\\ P\\ S \end{array} & \left(\begin{array}{ccc} -1 & -1-\alpha & -1+\alpha \\ -1+\alpha & -1 & -1-\alpha \\ -1-\alpha & -1+\alpha & -1 \end{array}\right), \end{array} \end{array}$$

and we show that a stepwise assembly into cyclic dominance, via the coexistence of two strategies, is possible if at least one of the intrinsic growth rates is sufficiently different.

This type of reasoning can be expanded to explore the assembly of cycles of any length, and even of more complex communities, such as those with higher-order interactions. The assembly of such communities has remained a challenging open question despite active recent research—both theoretical (28, 54) and empirical (19, 55)—showing the potential of such interactions to maintain ecological diversity.

## Concluding Thoughts

We argue that it is crucial for the field of evolutionary game theory to emphatically acknowledge that the replicator dynamics is more limiting than previously acknowledged (or fully appreciated) in terms of the biological systems that its predictions can apply to. Here, we have discussed a fundamental limitation of the replicator dynamics, the implications of which had not been reconsidered or explicitly acknowledged as a substantial caveat, even as the field moved to address other limitations, e.g., that the replicator dynamics ignores stochastic effects (15, 16, 47), that it muddles growth rates and interactions (56, 57), or that it apparently ignores more realistic genetics and demography (58). This fundamental assumption—that the game is the only source of fitness differences—is so likely to be broadly violated across natural systems, that the potential for paradoxical findings and unproductive debates is very high. Except where the replicator dynamics clearly applies or seems as suitable as any other available option [e.g., for the study of social behavior in humans (40, 59, 60)], it is high time that the field of evolutionary game theory fully embraces approaches that incorporate a more nuanced ecology, such as Lotka–Volterra or adaptive dynamics. And, in empirical systems that are more tractable (especially microbial experimental systems where one has more control over what goes in and what comes out), one could aim for still simple but less phenomenological theoretical models that allow for a more detailed analysis (61–63).

Given the limitations of the replicator dynamics, our paper adds further urgency to calls for the field of social behavior to undergo a serious—albeit admittedly nontrivial—reconsideration of terminology (43, 45, 64). Terms such as cooperation, altruism, public good, cheater, or free-rider have become both unproductively anthropomorphized and inextricably linked with game theoretic predictions. But the framework very likely does not apply as broadly as one encounters systems with costly behaviors that provide benefit to others. Furthermore, as we highlight in Fig. 2, the loose borrowing and translation of terms from games to ecological interactions can only amplify the confusion.

Although our focus here has been on the replicator dynamics in well-mixed populations, the same arguments apply to extensions of the framework to finite and/or structured populations, where the same assumption of identical intrinsic growth rates has been embraced. Reconsidering this body of work to probe the consequences of different intrinsic growth rates will likely lead to a productive rethinking of predictions, including in the realm of structured populations (17, 60). Conversely, because evolutionary game theory has developed sophisticated methods for thinking about a variety of complex interactions that only recently have been proposed—but remain understudied—in ecology (e.g., higher-order, spatially structured, multiscale, hierarchical, etc.), reestablishing the connection with ecological theory could substantially inform the latter in the study of complex species interactions.

## Supplementary Material

Appendix 01 (PDF)

## Data Availability

The data in the figures have been generated using Mathematica (65); the code can be found at doi: 10.5281/zenodo.15012169 (66). All other data are included in the article and/or *SI Appendix*.

## Footnotes

^*^A slightly different ecological approach consists of writing similar abundance equations but where the interaction effects are frequency-dependent, i.e. ${\dot{n}}_{i}=n_{i}\left(r_{i}+\sum_{j}a_{\mathrm{ij}}x_{j}\right)$. We address it in *SI Appendix* but the qualitative takeaways remain unchanged.

^†^A natural reaction is that, even when the system is bounded, if it is very large (e.g., as is the case in microbial populations) the problematic term in Eq. **3** might be negligible. This intuition, however, is incorrect because what matters is the magnitude of that term at the point where $f_{1}=f_{2}$ and, there, the fraction inside the brackets can still be nonnegligible when $r_{1}\neq r_{2}$. This can substantially change the coexistence fixed point and even its stability.

^‡^In fact, Hofbauer and Sigmund (29) demonstrated that the replicator dynamics for an **m-type game** is equivalent to the generalized Lotka–Volterra dynamics for an **(m-1)-type ecological system** when the intrinsic growth rates are different (e.g., a Prisoner’s Dilemma is equivalent to a 1-species population growth but not to a general predator–prey interaction).
