# Supplementary material for: Reconciling ecology and evolutionary game theory or “When not to think cooperation”
Source: Proc Natl Acad Sci U S A. 2025 Mar 31;122(14):e2413847122. doi: 10.1073/pnas.2413847122 (PMC12002174; doi:10.1073/pnas.2413847122)
Supplement: Supplementary file 1 — Appendix 01 (PDF) [file pnas.2413847122.sapp.pdf]

Supporting Information for

Reconciling ecology and evolutionary game theory or ‘When not to think cooperation’

Corina E. Tarnita, Arne Traulsen

Corina E. Tarnita, Arne Traulsen.
E-mail: ctarnita@princeton.edu, traulsen@evolbio.mpg.de

This PDF file includes:

Supporting text
Figs. S1 to S2
Table S1

Supporting Information Text

General number of types

For m types the Lotka-Volterra equations are

$$\dot{n}_i = n_i\big(r_i + \sum_{j=1}^{m} a_{ij} n_j\big) \quad [1]$$

where $i = 1, \ldots, m$. Then the frequency of individuals of type i is $x_i = n_i/n$, where $n = \sum_{k=1}^{m} n_k$ is the total population size. The dynamics of frequencies can be derived from the dynamics of abundances using the quotient rule:

$$\begin{aligned}\dot{x}_i &= \frac{\dot{n}_i n - n_i \dot{n}}{n^2} \\ &= \frac{n_i(r_i + \sum_{j=1}^{m} a_{ij} n_j)}{n} - \frac{n_i \sum_{k=1}^{m} n_k (r_k + \sum_{j=1}^{m} a_{kj} n_j)}{n^2} \\ &= x_i\Big(r_i + \sum_{j=1}^{m} a_{ij} n_j\Big) - x_i \sum_{k=1}^{m} x_k \Big(r_k + \sum_{j=1}^{m} a_{kj} n_j\Big) \\ &= n x_i \Big[\frac{r_i - \bar{r}}{n} + \sum_j a_{ij} x_j - \sum_{k,j} a_{kj} x_k x_j\Big],\end{aligned} \quad [2]$$

where $\bar{r} = \sum_k x_k r_k$ is the average intrinsic growth rate of the population and the two summation terms can be recognized as $f_i = \sum_j a_{ij} x_j$ (the fitness of type i) and $\bar{f} = \sum_k x_k f_k = \sum_{k,j} a_{kj} x_k x_j$ (the average fitness). Therefore, Eq. (2) can be rewritten as:

$$\dot{x}_i = \Big(\sum_{k=1}^{m} n_k\Big) x_i \left(\frac{r_i - \bar{r}}{\sum_{k=1}^{m} n_k} + f_i - \bar{f}\right) \quad [3]$$

When $m = 2$, Eq. (3) yields Eq. 3 in the main text. The term marked in red can be ignored only when the system is unbounded or the intrinsic growth rate of every type is equal to the average growth rate $\bar{r} = \sum_k r_k x_k$, which is equivalent to all types having identical intrinsic growth rates.

Frequency-dependent ecological interactions

For m types, the abundance equations with frequency-dependent interaction effects are

$$\dot{n}_i = n_i\big(r_i + \sum_{j=1}^{m} a_{ij} x_j\big) \quad [4]$$

where $i = 1, \ldots, m$. The frequency of individuals of type i can be derived as above using the quotient rule:

$$\dot{x}_i = x_i\Big[r_i - \bar{r} + \sum_j a_{ij} x_j - \sum_{k,j} a_{kj} x_k x_j\Big], \quad [5]$$

where $\bar{r} = \sum_k x_k r_k$ is the average intrinsic growth rate of the population and the two summation terms can be recognized as $f_i = \sum_j a_{ij} x_j$ (the fitness of type i) and $\bar{f} = \sum_k x_k f_k = \sum_{k,j} a_{kj} x_k x_j$ (the average fitness). Rewriting $r_i = r_i \sum_j x_j$ as well as $\bar{r} = \sum_{k,j} r_k x_k x_j$ and combining each a_{ij} term with an r_i, Eq. (5) becomes a replicator dynamics for the m-type game with payoff matrix $\tilde{A} = [r_i + a_{ij}]$:

$$\begin{aligned}\dot{x}_i &= x_i\Big[\sum_j (r_i + a_{ij}) x_j - \sum_{k,j} (r_k + a_{kj}) x_k x_j\Big] \\ &= x_i(\tilde{f}_i - \bar{\tilde{f}}_i).\end{aligned} \quad [6]$$

In other words, when the ecological interactions are frequency dependent, there is no 'problematic' term and there exists a direct transformation of the ecological system with m types into an m-type game whose matrix is modified to account for the payoffs. Although, at first glance, this might appear like good news, the fundamental problem of different intrinsic growth rates changing the outcome of the game remains. Specifically, let us for simplicity take the case $m = 2$: if one attempts an evolutionary game theoretical treatment for the m-type system with matrix A

$$A = \begin{pmatrix} a_{11} & a_{12} \\ a_{21} & a_{22} \end{pmatrix}$$

and thus, consistent with the setup of EGT, never asks oneself about the possibility of different intrinsic growth rates, then one would attempt to simply make predictions based on the matrix A. However, the correct predictions should be made for the transformed matrix

$$\tilde{A} = \begin{pmatrix} r_1 + a_{11} & r_1 + a_{12} \\ r_2 + a_{21} & r_2 + a_{22} \end{pmatrix}$$

and these predictions will differ. For instance, the correct condition that strategy A_1 is evolutionarily stable (ESS) would be $r_1 + a_{11} > r_2 + a_{21}$, which is not equivalent to $a_{11} > a_{21}$ when $r_1 \neq r_2$.

Possible transitions

Using asymmetric growth rates, we can transform the game between different scenarios, cf. Fig. 2 in the main text. Depending on the sign and the ranking of the payoffs, all possible transitions are shown in Fig. S1.

Step-wise assembly of a Rock-Paper-Scissors dynamics

Writing the 3-type Lotka-Volterra equations for the dynamics of abundances for the Rock-Paper-Scissors matrix in the main text, we obtain

$$\frac{d}{dt}n_R = n_R\left(r_R - n_R + (-1-\alpha)n_P + (-1+\alpha)n_S\right) \quad [7a]$$

$$\frac{d}{dt}n_P = n_P\left(r_P + (-1+\alpha)n_R - n_P + (-1-\alpha)n_S\right), \quad [7b]$$

$$\frac{d}{dt}n_S = n_S\left(r_S + (-1-\alpha)n_R + (-1+\alpha)n_P - n_S\right) \quad [7c]$$

where r_R, r_P, and r_S are the intrinsic growth rates of R, P, and S. The fixed points of this dynamics are

- Extinction of the population, $(n_R, n_P, n_S) = (0, 0, 0)$. Assuming positive growth rates, this fixed point is unstable
- Homogeneous populations, $(n_R, n_P, n_S) = (r_R, 0, 0)$, $(n_R, n_P, n_S) = (0, r_P, 0)$, $(n_R, n_P, n_S) = (0, 0, r_S)$. Positive growth rates ensure that these three fixed points are saddle points.
- Coexistence of two types (if the fixed point coordinates are positive).

  RP , $(n_R, n_P, n_S) = \left(\frac{r_R - r_P(1+\alpha)}{\alpha^2}, \frac{r_P - r_R(1-\alpha)}{\alpha^2}, 0\right)$

  PS $(n_R, n_P, n_S) = \left(0, \frac{r_P - r_S(1+\alpha)}{\alpha^2}, \frac{r_S - r_P(1-\alpha)}{\alpha^2}\right)$,

  RS $(n_R, n_P, n_S) = \left(\frac{r_R - r_S(1-\alpha)}{\alpha^2}, 0, \frac{r_S - r_R(1+\alpha)}{\alpha^2}\right)$.
- Finally, there is a fixed point RPS where all three types coexist (if its coordinates are positive) $(n_R, n_P, n_S) = \left(\frac{r_R\alpha - r_P(3-\alpha) + r_S(3+\alpha)}{9\alpha}, \frac{r_R(3+\alpha)\alpha + r_P\alpha - r_S(3-\alpha)}{9\alpha}, \frac{-r_R(3-\alpha) + r_P(3+\alpha) + r_S\alpha}{9\alpha}\right)$.

We first confirm that for $r = r_R = r_P = r_S > 0$ the dynamics has unstable fixed points at $(r, 0, 0)$, $(0, r, 0)$, and $(0, 0, r)$. In addition, there is—for symmetry reasons—a neutrally stable fixed point at $(r/3, r/3, r/3)$. Thus, when the three growth rates are the same, no two types can coexist but all three will coexist and cycle around the neutrally stable fixed point. The question is: Can this internal equilibrium be assembled pairwise, one mutant at a time, if not all growth rates are equal?

To answer this, we pick one of the three pairwise interactions, e.g., between Rock and Paper, and transform the outcome of the game, which would otherwise result in dominance of Paper, into coexistence by changing the growth rate r_R similar to the graphical illustration in Fig. 2B in the main text. We fix $r_P = r_S = r > 0$ and let $r_R = r + \rho$. To destabilize Paper, we need $r_R > (1+\alpha)r_P$, i.e. $\rho/r > \alpha$. In addition, we need to ensure that Rock alone remains unstable so that we can get coexistence, which is the case if either $\alpha \geq 1$ or if $\alpha < 1$ and $\rho/r < \alpha/(1-\alpha)$. With growth rates fulfilling these conditions, now Rock and Paper can coexist stably in the absence of Scissors at equilibrium abundances $n_R^* = (\rho - r\alpha)/\alpha^2$ and $n_P^* = (r\alpha - (1-\alpha)\rho)/\alpha^2$. Once Scissors arrives, either via mutation or via immigration, its initial growth rate is $3r + \rho - 3\rho/\alpha$. Scissors can invade only if this growth rate is positive, i.e. either if $\alpha \geq 3$ or if $\alpha < 3$ and $\rho/r < 3\alpha/(3-\alpha)$. Note that this last condition is always stronger than the condition to keep Rock unstable. Thus, the parameter range in which Scissors can invade the coexistence of Rock and Paper is narrower than the parameter range for a stable coexistence of Rock and Paper. The scenario of a stable coexistence and the possible invasion of the third type—holds for $\frac{3\alpha}{3-\alpha} > \rho/r > \alpha$ or $\rho/r > \alpha > 3$. Table S1 summarizes these conditions.

The only remaining questions are whether this newly assembled system still leads to stable coexistence of the three types and whether it still does so in a manner that reflects cyclical interactions. Specifically, the coexistence point needs to be either neutrally stable or stable and approached dynamically via dampened oscillations. A numerical sweep of the eigenvalues of the coexistence point confirmed the above conditions for the assembly, as well as the stability of the fixed point (Fig. S2). Moreover, we have also confirmed numerically that the imaginary eigenvalues have a negative real part when the equilibrium is stable and, thus, that the stable equilibrium is approached via dampened oscillations (Fig. S2).

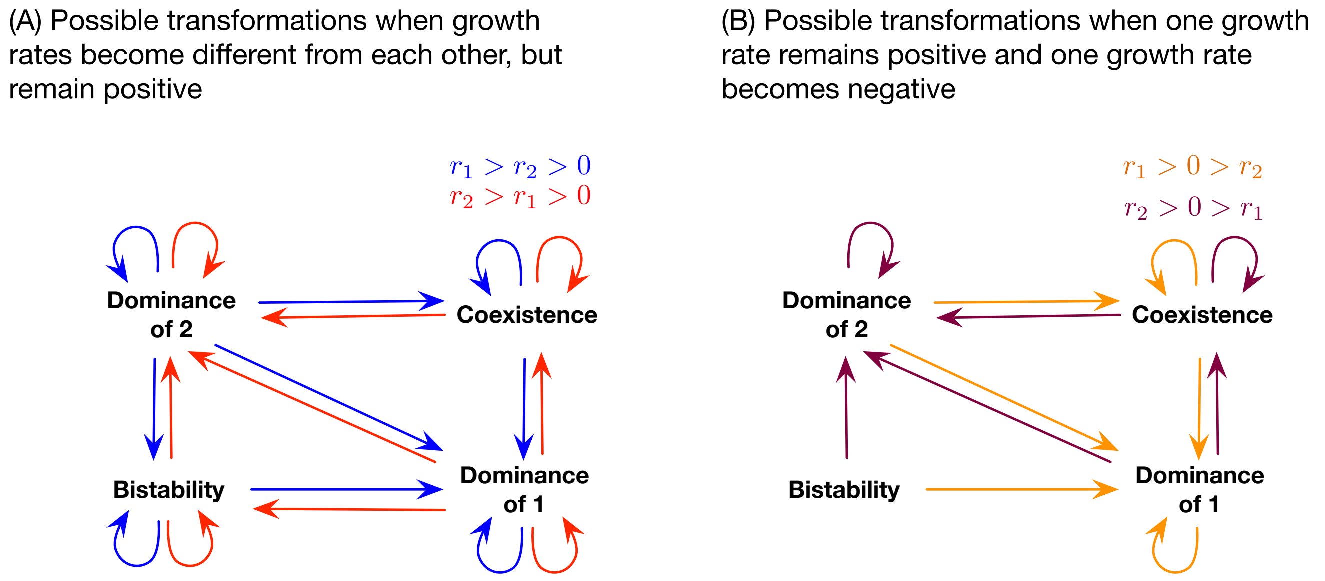

Fig. S1. **Possible transitions between stability scenarios** (A) Starting from symmetric growth rates, almost any stability transition can be achieved if one of the growth rates can be changed – even if growth rates remain positive. However, coexistences cannot become bistabilities (and vice versa). (B) When one growth rate becomes negative, the bi-stabilities vanish entirely and some dominances vanish as well.

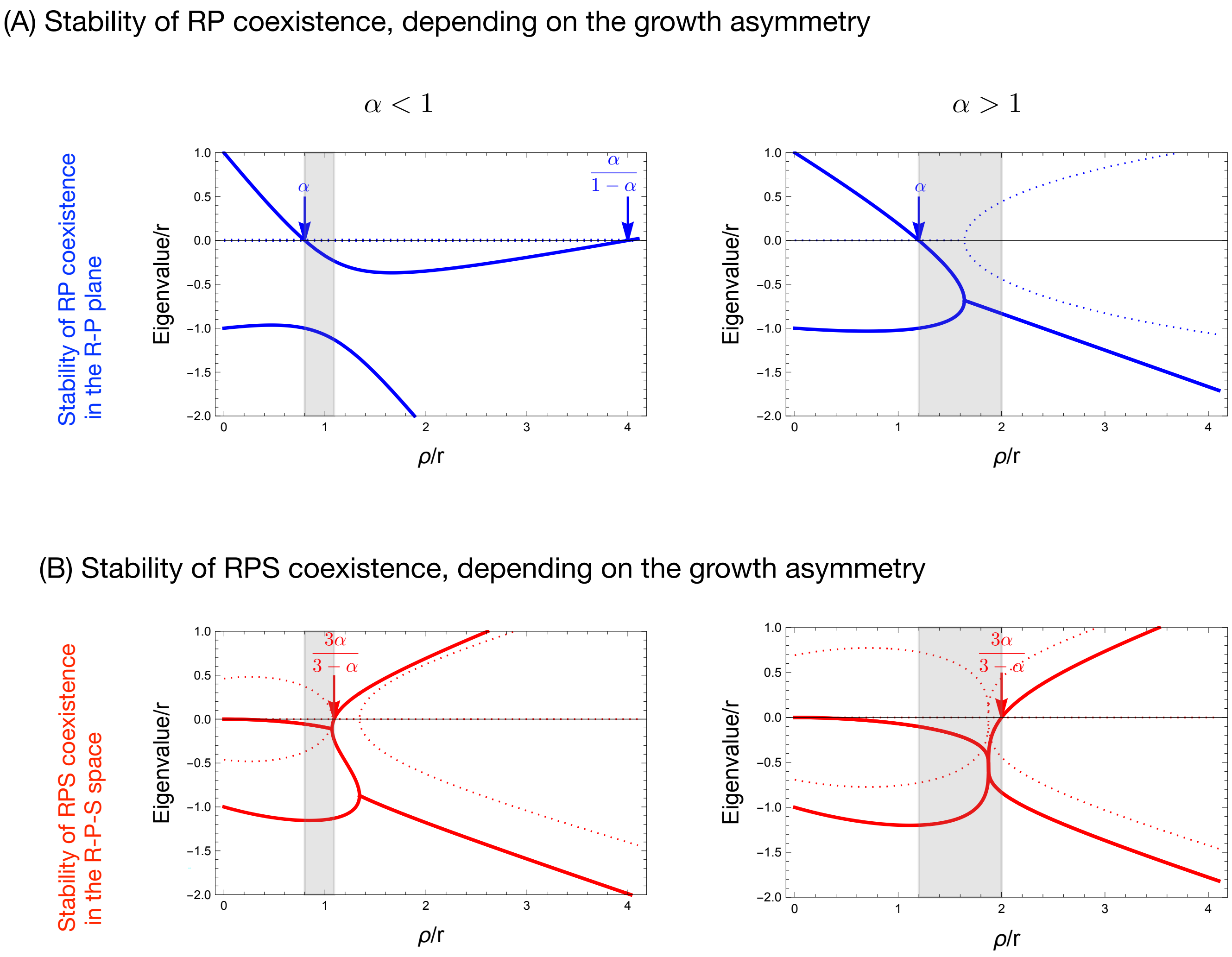

Fig. S2. **Stability in the rock paper scissors dynamics with asymmetric growth rates** We show a linear stability analysis of the fixed points with Rock and Paper only (A) and with Rock, Paper and Scissors (B), focusing on the Eigenvalues of the linearized system at the fixed points. Real parts of Eigenvalues are full lines, imaginary parts dotted lines. If all Eigenvalues have negative real parts, the corresponding fixed point is stable. Regardless of whether $\alpha < 1$ or $\alpha > 1$, there exists a subregion (marked in gray) for $\alpha < \frac{\rho}{r} < \frac{3\alpha}{3-\alpha}$ where Rock and Paper stably coexist, Scissors can invade, and the dynamics spirals into the RPS fixed point (Parameters $r = 1$, $\rho = 1.5$, $\alpha = 0.8$ in (A) and $\alpha = 1.3$ in (B)).

Table S1. When RP is stable in the absence of S and RPS is stable at the same time, we can gradually build up a coexistence of three types which shows cyclic dynamics. See Fig. S2 for a more detailed stability analysis.

	$0 < \alpha < 1$	$1 < \alpha < 3$	$3 < \alpha$
RP is stable	$\alpha < \frac{\rho}{r} < \frac{\alpha}{1-\alpha}$	$\alpha < \frac{\rho}{r}$	$\alpha < \frac{\rho}{r}$
RPS is stable	$\frac{\rho}{r} < \frac{3\alpha}{3-\alpha}$	$\frac{\rho}{r} < \frac{3\alpha}{3-\alpha}$	for all $\frac{\rho}{r}$
